# Long-term functional outcomes of diffuse pigmented villonodular synovitis of knee: The role of adjuvant radiotherapy

**DOI:** 10.1097/MD.0000000000023794

**Published:** 2021-03-26

**Authors:** Ju Chun Chien, Yi Ping Wei, Chun Yu Chen, Wei Hsin Hsiang, Yuan You Wang, Wen Shan Liu, Shan Wei Yang

**Affiliations:** a Department of Radiation Oncology; b Department of Orthopedics, Kaohsiung Veterans General Hospital, Kaohsiung; c Departement of Pharmacy, Tri-Service General Hospital Penghu Branch, Magong City, Penghu; d Department of Nursing, Kaohsiung Veterans General Hospital, Kaohsiung, Taiwan.

**Keywords:** functional outcome, pigmented villonodular synovitis, radiotherapy, synovectomy

## Abstract

Diffuse pigmented villonodular synovitis (PVNS) of knee is a rare benign disease that has a destructive clinical course. Synovectomy and adjuvant radiotherapy (RT) have been reported as treatment options but literatures reporting functional outcomes were sparse. This study aimed to evaluate the long-term functional outcomes and disease control among treatment modalities through the 22 years of experience.

A single-center database was searched for patients who received synovectomy of knee with the pathologic diagnosis of PVNS. General data, treatment modalities, and recurrent status were retrospectively collected from medical records. Functional outcomes were evaluated by Western Ontario and McMaster Universities Osteoarthritis Index through phone interviews by an independent orthopedist.

From January 1995 to December 2017, 24 patients with diffuse PVNS of knee were identified, including 19 receiving open synovectomy (OP) and 5 undergoing arthroscopic surgery. Adjuvant RT was performed on 14 patients with a median dose of 35 Gy (range 20–40 Gy). After median follow up of 6 years, recurrences were recorded in 10 cases. The recurrence rate was significantly lower in the OP + RT group than the OP group (8.3% vs 57.1%, *P* = .038). Among those with preserved knee joints, there was no significant difference in the Western Ontario and McMaster Universities Osteoarthritis Index score and stiffness score between patients in the OP + RT and OP groups.

For patients with diffuse PVNS of knee, the addition of moderate-dose adjuvant RT following OP provided excellent local control while maintaining good joint function with limited treatment-related morbidity. Our study emphasized the importance of moderate dose RT in diffuse PVNS of knee joint.

## Abstract 2

Supplemental Digital Content is available in the text

## 1 Introduction

Pigmented villonodular synovitis (PVNS) is a mono-articular proliferative process that originates from the synovial membranes and is also characterized by articular cartilage invasion. It is a rare sarcoma-like benign disease, and its incidence is about 1.8 cases per million people.^[1–3]^ Younger patients seem to be predominantly affected, and the incidence peaks at the age of 30 to 40 years.^[3]^

There are 2 distinct types of PVNS: localized and diffuse. The 2 types are histologically similar, but differ in the extent of synovial involvement. Diffuse PVNS presents with more pronounced symptoms and is more destructive. It has a high tendency to invade extra-articular structures, such as muscles, tendons, bones, neurovascular structures, and skin. Complete tumor removal may be challenging in patients with diffuse PVNS. The recurrence rates after surgical resection ranged from 8% to 56%, depending on the extent of surgery.^[4]^ Therefore, although classified as a benign disease, diffuse PVNS could cause severe disability at a relatively young age.

Despite having a high recurrence rate and possible severe joint function decline, there is a lack of strong evidence for the optimal treatment for diffuse PVNS.^[1,2]^ Open or arthroscopic synovectomy have been reported as treatment options for diffuse PVNS. According to previous studies, the recurrence rate is higher for diffuse PVNS patients receiving arthroscopic synovectomy when compared to those who undergo open synovectomy (OP).^[5–11]^ Furthermore, PVNS of different anatomical locations should be analyzed separately since it influences the choices of treatment and surgical techniques. Hence, surgical modalities and affected joints should be taken into consideration when comparing clinical outcomes of diffuse PVNS.

Adjuvant radiotherapy (RT) is also recognized as a treatment modality for diffuse PVNS. For the past decades, researches have focused on the disease control of diffuse PVNS with the addition of radiotherapy after synovectomy.^[12–15]^ Most of them are retrospective cohort studies with small sample sizes or even case reports. The results might be affected by confounding factors, such as localized versus diffuse types, affected joints, surgical modalities (open or arthroscopic synovectomy) and approaches (anterior only or anterior combined posterior approach), if no proper stratification or analytical correction was done with limited case numbers.^[14,15]^ Furthermore, the goals of PVNS treatment are not only preventing disease recurrence, but also relieving pain and stiffness, and preserving joint function. A recent cohort study reported the long-term functional outcomes for PVNS patients, but the result of stiffness was lacking.^[15]^ Therefore, the efficacy and long-term functional outcomes of adjuvant RT in treating diffuse PVNS of knee remained unclear.^[14,15]^

The purpose of the present study was to evaluate the disease control and long-term functional outcomes in treatments of diffuse PVNS of knee in a single medical center. Arthroscopic and OP with or without subsequent external-beam RT were compared. We hypothesized that OP with subsequent external-beam RT in treating diffuse PVNS of knee would lead to the less disease recurrence rate but more stiffness in the knee joint.

## 2 Methods

### 2.1 Data source and study subjects

After we received approval from the research ethics board, the pathology database of our institute was searched for patients who received synovectomy of knee with the pathologic diagnosis of PVNS. Those who had diffuse PVNS of knee joints were enrolled. PVNS originating from joints other than knee and localized PVNS were excluded. Clinical data were retrospectively collected from medical records, image studies, surgery notes, and radiotherapy charts. The data collected included the patient's age, sex, whether the disease was primary or recurrent, treatment modalities, radiation treatment technique and dose regimens, and current disease status.

### 2.2 PVNS type

Localized or diffuse PVNS was determined by pre-operative magnetic resonance imaging (MRI) or operative findings. The major features of the localized PVNS were localized nodules with a clear boundary and a single or no pedicle. In diffuse PVNS, the surface of the synovial membrane is villous, and there is an extensive involvement of the adjacent vessels and nerves.

### 2.3 Study endpoints

The primary endpoint was functional outcomes and the secondary endpoints included local control and skin reaction. Functional outcomes were evaluated by the standardized questionnaire of the Western Ontario and McMaster Universities Osteoarthritis Index (WOMAC score) through phone interviews by an independent orthopedist. The degree of hyperpigmentation, hypoalgesia, and any skin changes of the affected knee were scored by the RTOG (Radiation Therapy Oncology Group) scoring system.

The WOMAC score is widely used in the evaluation of hip and knee osteoarthritis and other hip and knee disabilities. It is a self-administered questionnaire consisting of 24 items that are divided into 3 subscales: pain, stiffness, and physical function. It is scored from 0 to 100, with higher the score denoting less perceived disability. The WOMAC score provides a more comprehensive assessment of patients’ subjective perception than the 4 functional categories assessments which was previously applied.^[15]^ It is also easier to understand than the Toronto Extremity Salvage score and functional rating systems of the Musculoskeletal Tumor Society, especially in conversations through a phone interview.^[15]^ Therefore, the WOMAC score was used.

Local control was defined as:

1) the absence of clinical evidence of disease (including joint effusion, swelling sensation, and locking of the joint) for at least 2 years after treatment or
2) stable disease in MRI imaging for at least 2 years after treatment.

### 2.4 Statistics

Data were processed and analyzed using the Statistical Package for the Social Sciences (SPSS, version 22.0, Armonk, NY; IBM Corp.). Considering the small sample size, nonparametric statistics with Fisher exact test was performed for categorical variable with a 95% confidence interval (CI). The mean WOMAC score of 90 was used to stratify the study cohort for analysis (WOMAC > 90 vs WOMAC < = 90). A mean stiffness score of 6 was used as a cut-off (stiffness score > 6 vs < = 6). The correlation between WOMAC score and RT dose as continuous variables was evaluated by logistic regression.

## 3 Results

### 3.1 Study population and treatment modalities

From 1995 to 2017, 40 consecutive histologically proven cases of PVNS were found. Seven cases were excluded due to localized PVNS, and 9 were excluded due to origins other than knee joint. Twenty-four patients with diffuse PVNS of knee were included, including 13 women and 11 men. The mean age was 40.4 years (range 19–65 years) (Table 1). Of the 24 patients, the majority were treatment-naïve without previous surgical interventions or RT before the index operations to the affected knee. Only 1 patient had undergone a previous synovectomy.

**Table 1:** Patient and disease demographics.

Characteristic	No. of Patients (%)
Gender	
Men/ female	11 (45.8%)/ 13 (54.2%)
Mean age (range)	40.4 [19–65]
Presenting status	
Primary	23
Local recurrence	1
Disease recurrence	
Image proof	6 (1 patient received MRI at other hospital)
Clinical symptoms	4 (3 of them received the total joint arthroplasty)
No evidence of recurrence	14
Treatment	
OP alone	7
OP and postoperative RT (OP + RT)	12
AS alone	3
AS and postoperative RT (AS + RT)	2

AS = arthroscopic surgery, OP = open synovectomy, RT = radiotherapy.

Regarding the surgical modalities, the decisions between arthroscopic or OP were based on surgeon's clinical judgment and patients’ preference, considering the coverage of health insurance. Open and arthroscopic synovectomy were performed in 19 and 5 patients, respectively (Table 1). For the approach of synovectomy, posterior synovectomy was not a routine procedure for diffused PVNS in our hospital. The choice between anterior approach with or without posterior approach of synovectomy was mainly based on pre-operative MRI finding. Combined posterior approach of synovectomy was adapted when the anterior or posterior cruciate ligament was extensively invaded by PVNS. Among the study cohort, there were 7 cases receiving anterior combined posterior approaches of open synovectomies. There was no case of combined anterior arthroscopic and posterior OP.

Surgical finding, surgeon's opinion, and patient's preference were taken into consideration for the decisions on radiotherapy. Adjuvant radiotherapy were done in 58.3% (14/24) of the cases (Table 1), with 3-dimensional (3D) conformal or intensity-modulated radiation therapy technique. The median RT dose was 35 Gy (range, 20–40 Gy) over a median of 16 fractions (range, 10–20 fractions). The most applied RT regimen was 30 to 36 Gy over 14 to 18 fractions (n = 11, Table 2).

**Table 2:** Radiotherapy delivered.

Treatment Modality	Dose, Gy	No. of fractions	No. of patients	No. of recurrence	WOMAC score (total = 100)	Stiffness score in WOMAC (total = 8)	RTOG grading for skin reaction
EBRT on knee	0	0	10	6	48, 57, 82, 93, 95, 100^∗^	4, 8, 8, 6, 4, 0	
	20	10	1	1	100	0	0
	25	10	1	1	89	6	0
	30	15	2	0	99, 90	7, 6	0, 0
	34	17	1	0	98	6	0
	34.5	15	1	0	98	6	1
	35	14	2	0	80,75	4,8	1,1
	36	18	5	1	97,98,98,79^†^	6, 8, 7, 6	1, 0, 0, 1
	40	20	1	0	96	6	0

The correlation between WOMAC score and RT dose was checked by logistic regression, and showed no significant finding. (*P* = .577). EBRT = external-beam radiotherapy WOMAC = Western Ontario and McMaster Universities Osteoarthritis Index.

∗ Four of the 10 patients finally received the total joint arthroplasty of the affected joints because of progressive osteoarthritis post-treatment.

† One of the 5 patients refused to complete the WOMAC score on the telephone access, and the patient denied suffering from the clinical symptoms (joint effusion, swelling sensation, and locking of the joint) after treatment.

No patient received intra-articular yttrium-90, monocloncal antibodies, nor tyrosine kinase inhibitor.

### 3.2 Local recurrence

After a median follow-up of 6 years (range 2–15 years), the overall local recurrence rate was 41.7% (10 of 24 patients), including 6 demonstrating progressive gross recurrent disease on post-treatment MRI and 5 pathologically proved recurrence with re-operation of the affected knee. Comparing the outcomes among treatment modalities, there was a significantly higher local control rate with OP and adjuvant RT (Supplementary Table 1, http://links.lww.com/MD/F435). When focusing on those receiving OP, the local control benefit of adjuvant RT was still apparent, with a local recurrence rate of 8.3% in the OP + RT group versus 57.1% in the OP group (*P *= .038) (Table 3).

**Table 3:** Efficacy and functional outcome of the Four treatment modalities.

Treatment Modality	Local recurrence rate	Mean WOMAC score of the affected knee joint (range)	Mean stiffness score in WOMAC score (range)	Fisher *P* value
OP + RT	1/12	N = 11 91.6 (79–99)	6.5 (4–8)	.038^‡^
OP	4/7	N = 4^∗^ 84 (48–100)	5.5 (4–8)	
AS	3/3	N = 2^†^ 69.5 (57–82)	8	
AS + RT	2/2	N = 2 94.5 (89–100)	7 (6–8)	

AS = arthroscopic surgery, OP = open synovectomy, RT = radiotherapy, WOMAC = Western Ontario and McMaster Universities Osteoarthritis Index.

∗ Three of the 7 patients finally received the total joint arthroplasty of the affected joints.

† One of the 3 patients finally received the total joint arthroplasty of the affected joints.

‡ Comparison OP group with OP + RT group in local recurrence at follow-up by Fisher test.

### 3.3 Adverse effects

With moderate radiation dose and the use of 3D conformal or intensity-modulated radiation therapy technique, the acute and long-term toxicities of adjuvant radiotherapy were relatively mild in our study. There was no case of surgical wound breakdown nor deep wound infection recorded in those receiving adjuvant RT. Furthermore, there was no grade 2 or greater long-term skin reaction nor soft tissue edema reported.

### 3.4 Overall functional outcomes

Among the 24 cases, 4 patients were excluded from WOMAC score evaluation due to receiving total knee arthroplasty (TKA) of the affected joints for progressive osteoarthritis. Three of the 4 patients were in the OP group and 1 was in the arthroscopic synovectomy group, all without adjuvant radiotherapy.

There were 20 patients with preserved knee joints at recruitment. Nineteen of them completed the functional outcome measures through phone interviews while 1 refused the interview. After long-term follow-up, the mean WOMAC score was 88 (range 48–100) (Tables 3 and 4). An extremely poor functional outcome was reported in a 59-year-old female (WOMAC score = 48). She first received open anterior and posterior synovectomy without adjuvant RT. However, local recurrence was noted and she underwent re-operation in other hospital. According to her statement, there were difficulties going up and down the stairs, as well as arising from a sitting position. She also reported persistent joint pain and stiffness at the 5-year post-operative follow-up with a WOMAC stiffness score of 4, WOMAC pain score of 9, and WOMAC physical function score of 35.

**Table 4:** Comparison open synovectomy with open synovectomy  + = radiotherapy in functional outcomes.

					WOMAC score			Stiffness score in WOMAC		
Treatment Modality	Approach	No. of patients	WOMAC score	Stiffness score in WOMAC	>90	< = 90	Fisher *P* value	>6	< = 6	Fisher *P* value
OP + RT	Anterior approach	5	79, 80, 96, 98, 98	6, 4, 6, 7, 8						
	Anterior and Posterior approach	7^∗^	75, 90, 97, 98, 98, 99	8, 6, 6, 6, 8, 7	7/11	4/11	1.000	5/11	6/11	.604
OP	Anterior approach	3^†^	100	7						
										
	Anterior and Posterior approach	4^‡^	48, 93, 95	4, 6, 4	3/4	1/4		1/4	3/4	

OP = open synovectomy, RT = radiotherapy, WOMAC = Western Ontario and McMaster Universities Osteoarthritis Index.

∗ One patient refused to complete the WOMAC score.

† Two patient finally received the total joint arthroplasty.

‡ One patient finally received the total joint arthroplasty.

To analyze the impact of adjuvant RT and disease recurrence on joint function, further analysis was performed, excluding those who received TKA. The proportion of patients with good functional outcomes (WOMAC score > 90) was higher among those without recurrent disease (8/12 without recurrent PVNS versus 3/7 for those with recurrent PVNS). However, the correlation between recurrence and poor WOMAC score was not statistically significant (*P* = .226). When testing the effect of adjuvant RT, rates for good functional outcomes were 61.5% (8/13) for those receiving adjuvant RT, which was higher than 50% (3/6) for those without adjuvant RT, though no statistically significant difference found. Regression between the WOMAC score as continuous variable and the RT dose revealed no significant correlation (Table 2). Neither the total WOMAC score nor stiffness score was significantly different between those who received adjuvant RT and those who did not. These were both observed in the overall study population and the OP group (Table 4, Supplementary Table 1, http://links.lww.com/MD/F435).

## 4 Discussion

Focusing on diffuse PVNS of knee joint, the main findings of our study were

1) arthroscopic synovectomy resulted in a higher recurrence rate than OP;
2) adjuvant RT might improve disease control, which was also found for those who underwent OP; and
3) after long-term follow-up, adjuvant RT was not associated with worse stiffness or WOMAC functional outcomes, which is also a new contribution to current literatures.

In previous studies, arthroscopic synovectomy was linked with higher PVNS recurrence rate when comparing to OP. An observational study published in 2011 indicated that there was limited access to the affected joint during arthroscopic surgery in 52.9% (9/17 cases) of patients.^[14]^ Blanco et al has reported that arthroscopic synovectomy alone was insufficient to eliminate all affected tissue.^[10]^ In another retrospective cohort study published in 2015, recurrence rate of 36% (35/97) in open, 58% (69/118) in arthroscopic, and 50% (5/10) in arthroscopically-assisted mini OP were reported, which was consistent with the finding in our study.^[11]^ These literatures support the necessity of evaluating the effects of different surgical modalities. However, limited studies had compared treatment outcomes with stratification by the surgical modalities.^[5,11–15]^ Having such different recurrence rate, the surgical modalities should be taken into consideration when assessing other treatment options.

Adjuvant radiotherapy has long been recognized as an important treatment modality for PVNS to lower the rate of disease recurrence.^[12,13]^ In the latest meta-analysis including 35 retrospective cohort studies, the addition of adjuvant RT following synovectomy significantly decreased the disease recurrence rate.^[16]^ The benefit was observed both in patients receiving open and arthroscopic synovectomy; (disease recurrence rate, RT vs. no RT, overall: 11.4% vs. 36.9%, *P*< .001; OP: 10.9% vs. 31.3%, *P* = .002; arthroscopic synovectomy: 11.6% vs. 51.0%, *P* < .001). When focusing on diffuse PVNS of knee, our study also revealed a superior disease control with adjuvant RT, both in the overall study population and the OP subgroup.

Despite the well-recognized disease control advantage of adjuvant RT, there is no consensus on the radiation dose and regimen at present. Berger et al reported 7 patients who were treated with 30 to 50 Gy post-operatively.^[12]^ All of them had their disease controlled without late complications after 29 months of follow up.^[12]^ Park et al reported that the effect of low-dose radiation therapy at 20 Gy was similar to that of a moderate dose about 35 Gy.^[14]^ Griffin et al used RT with a mean dose of 39.8 Gy (range 24–50 Gy in 13–25 fractions).^[15]^ After the mean follow up of 94 months, the mostly applied regimen of 35 Gy over 14 fractions led to an 88.5% local control, while the disease control rate being 82.0% for the whole study cohort.^[15]^ Therefore, it is believed that a low to moderate dose of adjuvant RT could achieve a satisfactory local control rate.

The goal of PVNS treatment is not only to prevent disease recurrence, but also to preserve joint function and quality of life. Griffin et al had assessed the efficacy and long-term functional outcomes with adjuvant RT in 50 diffuse PVNS cases, including 60% of which having multiple surgeries before receiving RT.^[15]^ The local control rate was 94%. However, 4 out of the 14 patients who completed functional evaluation were rated as having poor joint function, leaving some concerns about late tissue reaction of RT.^[15]^ Long-term functional outcome was also reported in a meta-analysis with a mean follow up of 56 months.^[16]^ The proportion of patients experiencing stiffness of the affected knee was 3.3% for those receiving RT and was 8.0% for those without RT (*P* = .068).^[16]^

Similarly, having 95% of those with preserved knee joints completing functional evaluation after the median follow up of 72 months, our cohort indicated that there was no significant difference in the rate of joint stiffness nor functional outcomes between patient with and without adjuvant RT. Furthermore, the results revealed a non-significant decrease in stiffness and poor activity with RT, which are contrary to previous understanding. This might be explained by the reduced recurrence rate and therefore preventing soft tissue scarring from multiple surgeries. Though no significant association observed between PVNS recurrence and lower WOMAC score (*P* = 0 .226; Table 5.), the lack of statistical proof of the association might be caused by the small sample size and possible selection bias. By excluding the 4 patients having the worse clinical conditions who received TKA, the analytic power of association between recurrence and poor function might be weakened. Other outcomes including joint pain, physical function, limbs edema, and chronic skin reaction were also evaluated without evident inferiority found with adjuvant RT. The non-inferiority was observed in both overall study cohort and those receiving OP. Addition of radiotherapy did not appear to worsen joint function and the poor functional outcomes reported by Griffin et al might be the result of exposure to multiple surgeries.

**Table 5:** The correlation between recurrence and Western Ontario and McMaster Universities Osteoarthritis Index score.

		WOMAC score		
		< = 90	>90	Total
Recurrence				
	No	4	8	12
	Yes	4	3	7
Total		8	11	19

Exact significance: 0.226. WOMAC = Western Ontario and McMaster Universities Osteoarthritis Index.

Comparing to previous literatures, our study focused on the functional outcomes of patients with diffuse PVNS of knee.^[5,11–15]^ The functional outcomes were not limited to joint stiffness, but also joint pain, physical function, and soft tissue reaction of the involved knee joint. The impact of RT on disease control and functional outcomes were both analyzed with stratification by surgical modalities. Nevertheless, there were some limitations in our study. First, the small sample size due to the rarity of PVNS might compromise the statistical power. Second, the decisions on surgical modalities and the use of radiotherapy might cause selection bias in the retrospective observational study. Since the best disease control without worsen functional outcomes occurred in the most extensively treated subgroup (OP + RT), the local control advantage and the safety of adjuvant RT would still be reliable. Third, new treatment options such as monoclonal antibodies and tyrosine kinase inhibitors were not included in the treatment of our PVNS cohort since they were not covered by the national health insurance. Therefore, the validity of our result might be limited when extrapolating to patients receiving such target therapies. Further research with larger cohort and inclusion of more contemporary treatments is warrant.

## 5 Conclusion

Focusing on diffuse PVNS of knee, the addition of moderate-dose adjuvant RT following OP provided excellent local control. There was no significant treatment-related morbidity and the functional outcomes were not negatively affected by radiotherapy.

## Author contributions

**Conceptualization:** Chun Yu Chen.

**Data curation:** Chun Yu Chen, Wen Shan Liu, Shan Wei Yang.

**Supervision:** Wen Shan Liu, Shan Wei Yang.

**Writing – original draft:** Ju Chun Chien, Yi Ping Wei.

**Writing – review & editing:** Wei Hsin Hsiang, Yuan You Wang, Wen Shan Liu, Shan Wei Yang.
